# Supplementary material for: Order-of-magnitude enhancement in photocurrent generation of Synechocystis sp. PCC 6803 by outer membrane deprivation
Source: Nat Commun. 2022 Jun 2;13:3067. doi: 10.1038/s41467-022-30764-z (PMC9163127; doi:10.1038/s41467-022-30764-z)
Supplement: Supplementary file 1 — Supplementary Information [file 41467_2022_30764_MOESM1_ESM.pdf]

Order-of-magnitude enhancement in photocurrent generation of *Synechocystis* sp. PCC 6803 by outer membrane deprivation

Kusama *et al*.

(a)

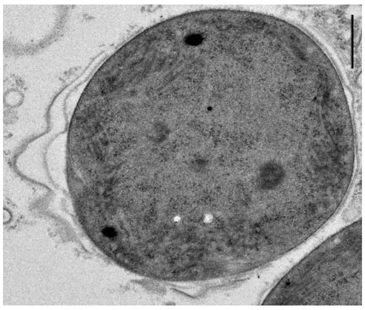

(b)

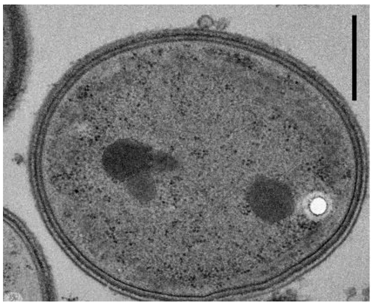

(c)

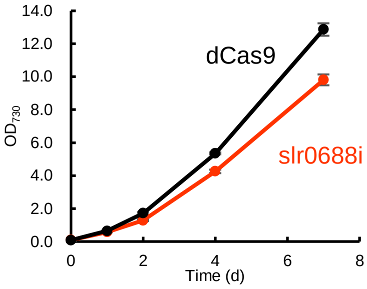

Supplementary Figure 1. Phenotypes of slr0688i strain. (a, b) Electron micrographs of ultra-thin sectioned slr0688i (a) and dCas9 (b) cells. Bars, 500 nm. These are the representative micrographs obtained by the observation of three biologically independent cell preparations. (c) Growth curves of slr0688i (red) and dCas9 (black) cells. Presented are average values of three biological replicates ± SD. Source data are provided as a Source Data file.

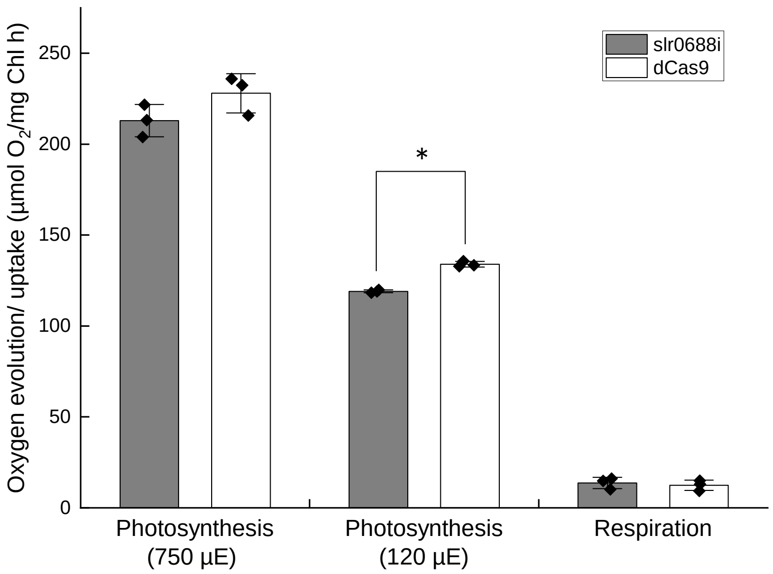

Supplementary Figure 2. Photosynthetic and respiratory activity of slr0688i and dCas9. Rates of photosynthesis and respiration of slr0688i (gray bars) and dCas9 (white bars) are expressed as oxygen evolution and uptake, respectively. Presented are all the data points obtained (black diamonds) as well as average values of three biological replicates ± SD. An asterisk indicates a statistically significant difference (two-sided *t* test; $p = 0.0001$). Source data are provided as a Source Data file.

(a)

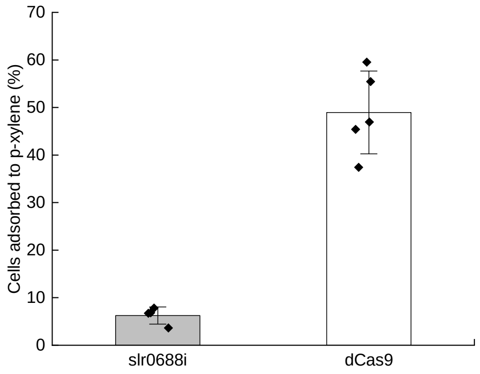

(b)

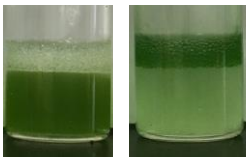

Supplementary Figure 3. Hydrophobicity of the cell surface of slr0688i and dCas9.

(a) Hydrophobicity of the cell surface of slr0688i and dCas9 was evaluated by Microbial adhesion to hydrocarbons assay. Percentage of the cells adsorbed to hydrocarbon was calculated as [{(A_{730} before *p*-xylene addition) − (A_{730} of aqueous phase after mixed with *p*-xylene)} × 100] / (A_{730} before *p*-xylene addition). Presented are all the data points obtained (black diamonds) as well as average values ± SD (slr0688i, n=4; dCas9, n = 5). (b) Photographs of cell suspensions mixed with *p*-xylene. Aqueous phase is located below *p*-xylene phase. Left, slr0688i; right, dCas9. Source data are provided as a Source Data file.

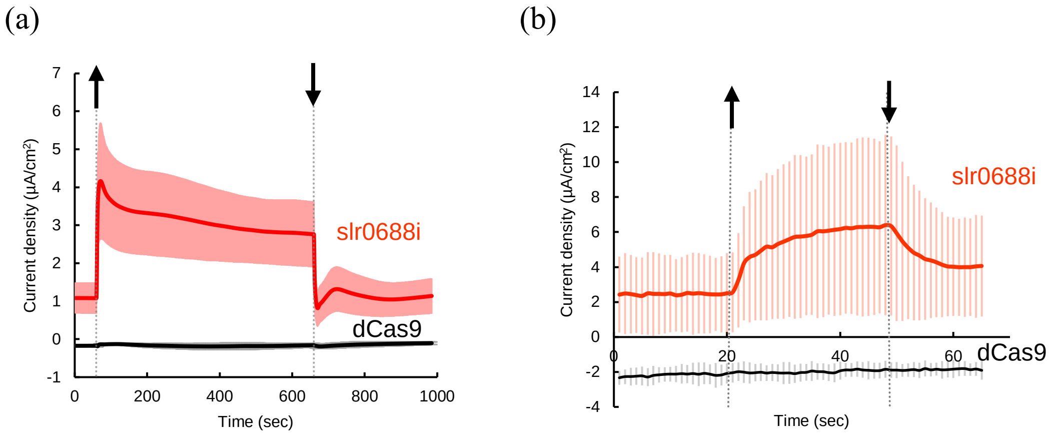

Supplementary Figure 4. Photocurrent generation from slr0688i cells in the presence of an artificial mediator, ferricyanide. (a) Slr0688i and dCas9 (OD_{730} = 2.0, 4 mL) cells after 3 to 4 days of culture resuspended in fresh BG11 medium with 1 mM ferricyanide (red and black line, respectively) were injected by gravity onto flat ITO and +0.28 V vs Ag/AgCl was applied. Averages ± 2 SE from 3 biological replicates are presented. (b) Slr0688i and dCas9 (OD_{730} = 30, 2 mL) cell suspensions after 3 days of culture resuspended in fresh BG11 medium containing 1 mM ferricyanide (red and black line, respectively) were stirred by Ar bubbling (flow rate, 104 ml min^{-1}) during measurements, and +0.28 V vs Ag/AgCl was applied to flat ITO anodes. Averages ± 2 SE from 3 biological replicates are presented. The upward and downward arrowheads indicate the beginning and end of illumination (420 µmol photons m^{-2} s^{-1}), respectively. Source data are provided as a Source Data file.

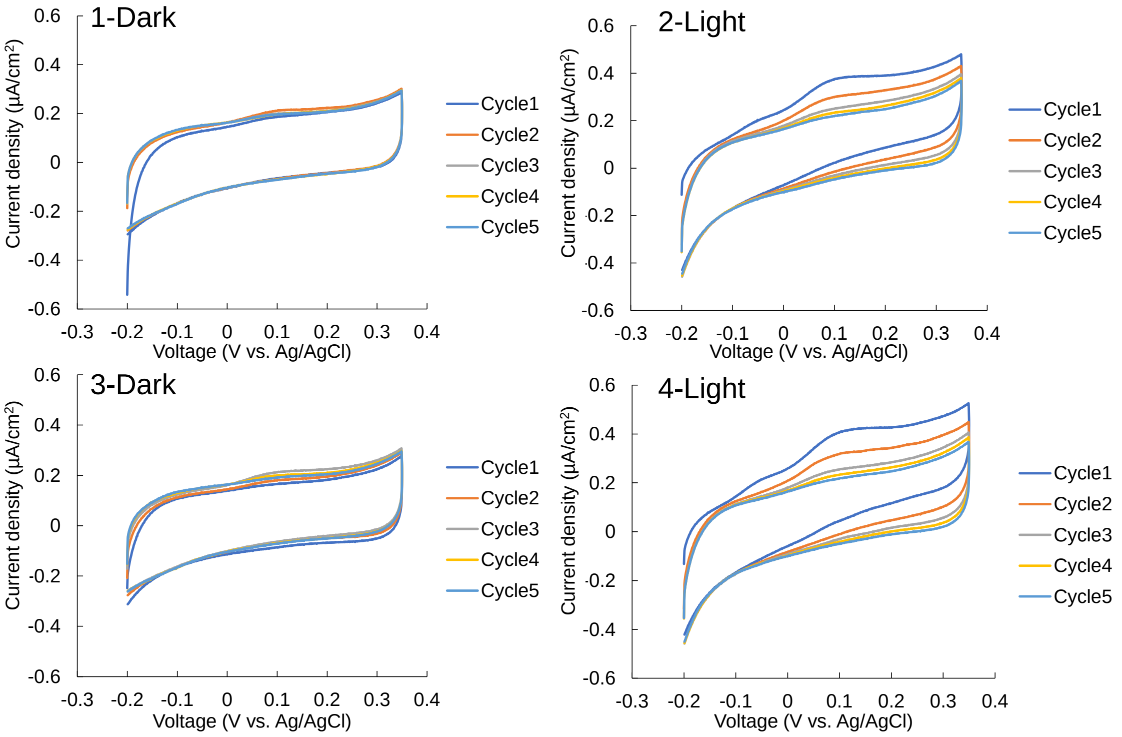

Supplementary Figure 5. Cyclic voltammograms of slr0688i in the dark and under illumination (100 µmol photons m^{-2} s^{-1}). Slr0688i cells (OD_{730}=1.4; orange line) were injected by gravity on plane ITO electrodes, followed by sequential cyclic voltammetry measurements (1-Dark→2-Light→3-Dark→4-Light). Scan rate, 6 mV/sec. Source data are provided as a Source Data file.

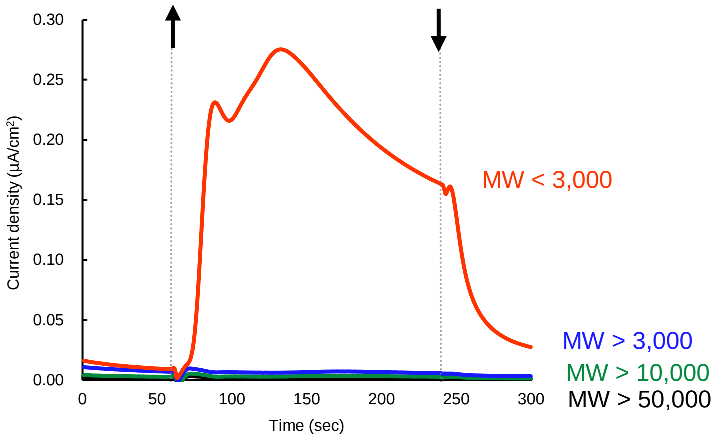

Supplementary Figure 6. Generation of photocurrent from slr0688i suspended in size-fractionated supernatants. Slr0688i cells (OD_{730} = 2.1) were collected and resuspended in 0.1× concentration of supernatants containing compounds with molecular weight > 50,000 (black), > 10,000 (green), > 3,000 (blue) and < 3,000 (red). For details, see Methods. +0.25 V vs Ag/AgCl was applied. The upward and downward arrows indicate the beginning and the end of illumination (120 µmol photons m^{-2} s^{-1}), respectively. Source data are provided as a Source Data file.

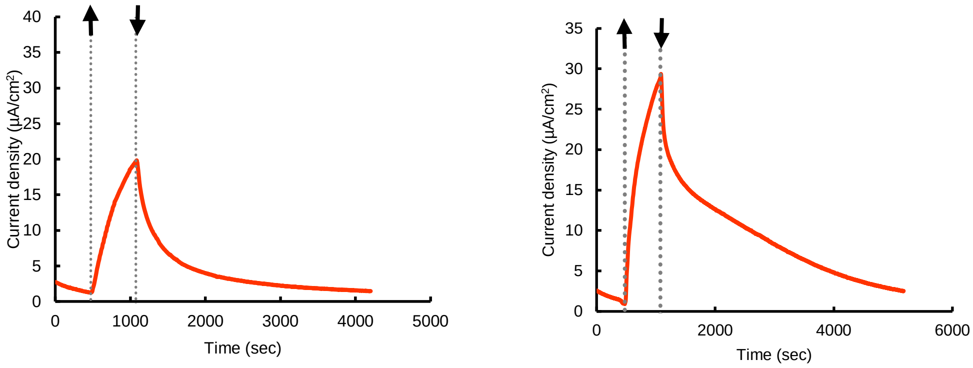

Supplementary Figure 7. Photocurrent from slr0688i with CP anodes. Shown are biological replicates of Fig. 1e. Slr0688i (OD_{730} = 14.6, 4 mL) after 6 days of culture were injected by gravity onto a piece of carbon paper placed upon flat ITO, and +0.25 V vs Ag/AgCl was applied. The upward and downward arrowheads indicate the beginning and end of illumination (420 µmol photons m^{-2} s^{-1}), respectively. Source data are provided as a Source Data file.

(a)

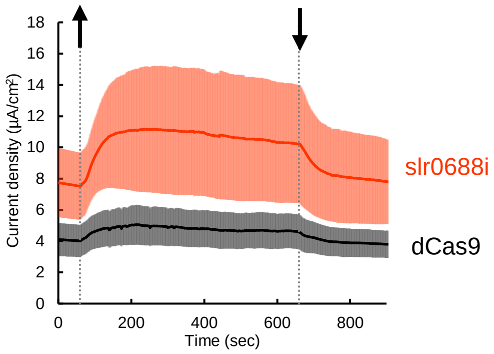

(b)

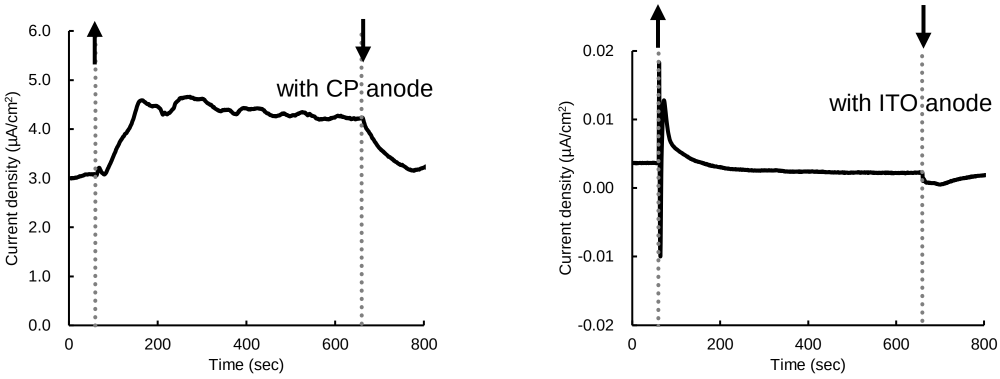

Supplementary Figure 8. Characterization of photocurrent generation from slr0688i cells with a CP anode. (a) Comparison of photocurrent generated by slr0688i cells to that by dCas9 cells. Slr0688i and dCas9 cells (OD_{730} = 2.0, 4 mL) after 3 days of culture in respective supernatants were injected by gravity onto a piece of carbon paper placed upon plane ITO, and +0.25 V vs Ag/AgCl was applied. Averages ± 2 SE from 10 and 9 biological replicates for slr0688i and dCas9, respectively, are presented. Note that the cells of mid-exponential growth phase were used for these comparative photocurrent measurements, as the cells of this growth phase tended to show more reproducible photocurrent levels than those of the cells of late-exponential or early stationary growth phase. (b) The effect of replacement of the culture supernatant to fresh BG11 medium. Slr0688i (OD_{730} = 2.0, 4 mL) cells after 3 days of culture resuspended in fresh BG11 were

injected by gravity onto either a piece of carbon paper (left) or plane ITO (right), to which +0.28 V vs Ag/AgCl was applied. The upward and downward arrowheads indicate the beginning and end of illumination (420 μmol photons m^{-2} s^{-1}), respectively. Source data are provided as a Source Data file.

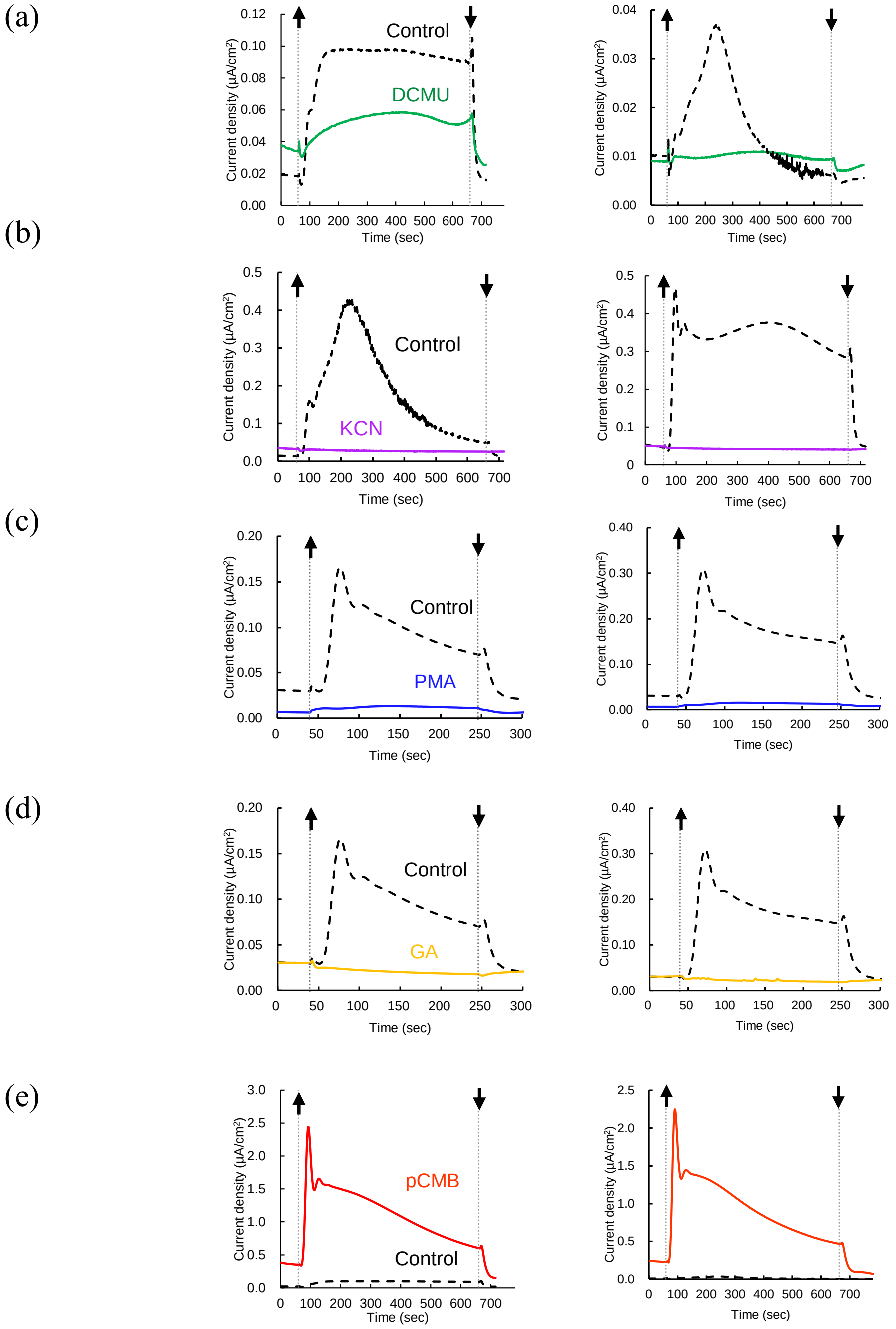

Supplementary Figure 9. Effects of photosynthesis inhibitors on current generation from slr0688i. Shown are biological replicates of Fig. 2a; current generation from slr0688i cells treated with (a) 10 µM DCMU (green), (b) 5mM KCN (violet), (c) 50 µM PMA (blue), (d) 10 mM GA (yellow) and (e) 100 µM pCMB (red) at +0.25 V vs. Ag/AgCl are shown. Black dashed lines are the control photocurrents obtained from cell suspensions incubated with respective volumes of solvents without inhibitors. The upward and downward arrows indicate the beginning and the end of illumination (70 µmol photons m^{-2} s^{-1}), respectively. Source data are provided as a Source Data file.

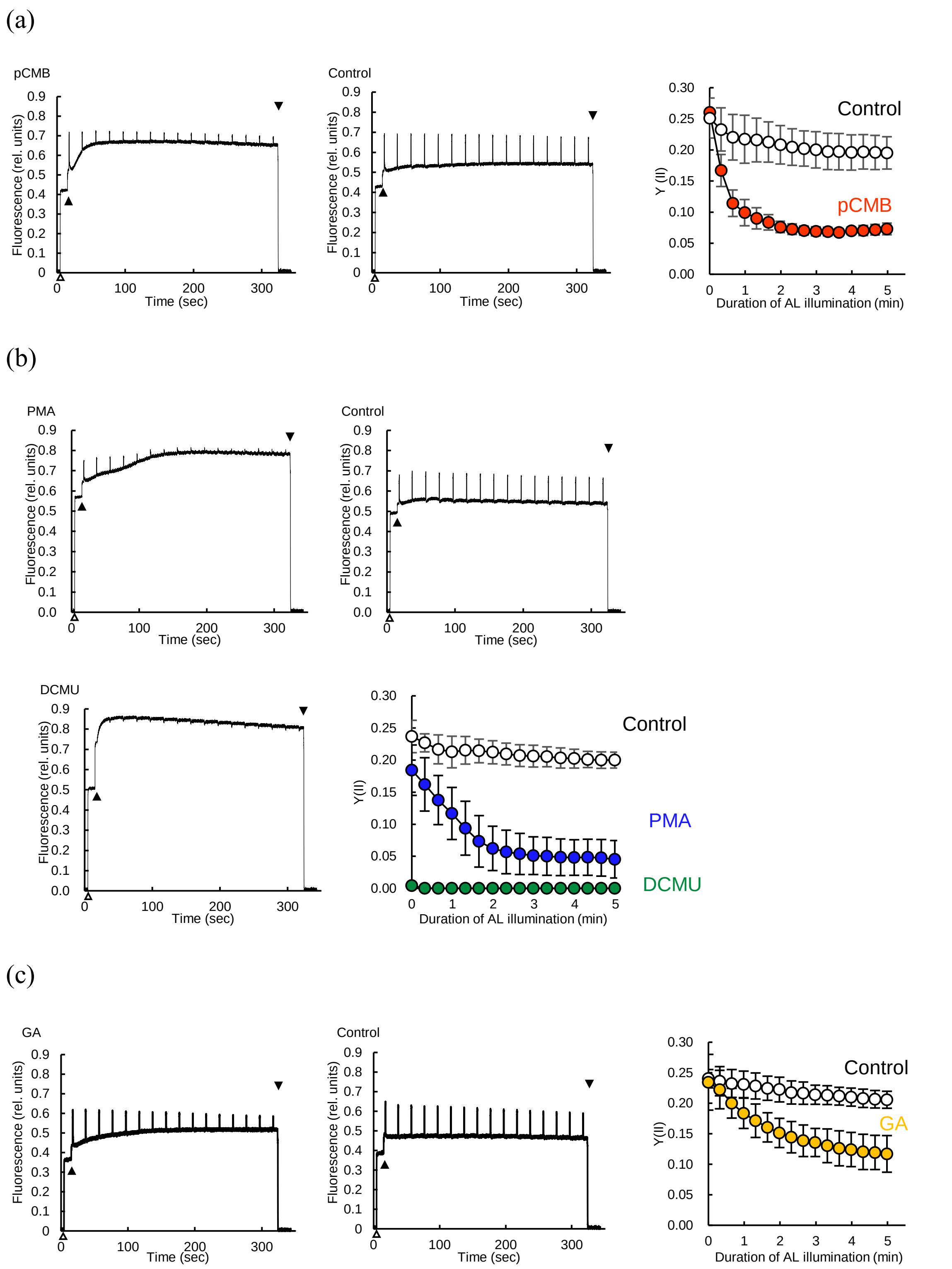

Supplementary Figure 10. Effects of photosynthesis inhibitors on chlorophyll fluorescence yields. Slr0688i cells (OD_{730}=13) treated with (a) 100 μM pCMB (red), (b)

10 μM DCMU (green), 50 μM PMA (blue), and (c) 10 mM GA (yellow) were used for chlorophyll fluorescence measurements. Also shown are the control datasets obtained with cell suspensions incubated with respective volumes of solvents without inhibitors (white). Representative kinetics of chlorophyll fluorescence and changes in effective quantum yield of PSII (average values of three biological replicates ± SD) are shown. The onset of the measuring light illumination is indicated by white upward triangles, and the beginning and the end of the actinic light illumination are indicated by upward and downward black arrows, respectively. Source data are provided as a Source Data file.

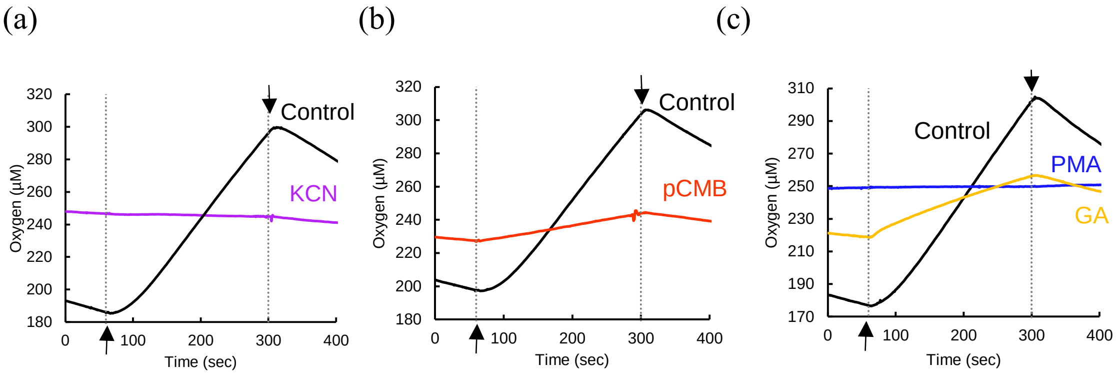

Supplementary Figure 11. Effects of photosynthesis inhibitors on whole chain electron transport activity of slr0688i. Oxygen evolving activity was measured in the presence of 5 mM $NaHCO_3$ with oxygen electrode. (a) 5 mM KCN (violet) was added to slr0688i cells (12 µg chl/ml) just before the measurement. As to other inhibitors, slr0688i cells (24 µg chl/ml) were treated with (b) 100 µM pCMB (red) for 1.5 h, (c) 50 µM PMA (blue) or 10 mM GA (yellow) for 30 min, followed by dilution to 12 µg chl/ml and measurements of oxygen evolving activity. Respective controls, which were treated only with respective solvents, are shown in black. The beginning and the end of illumination are indicated by upward and downward arrows, respectively. Source data are provided as a Source Data file.

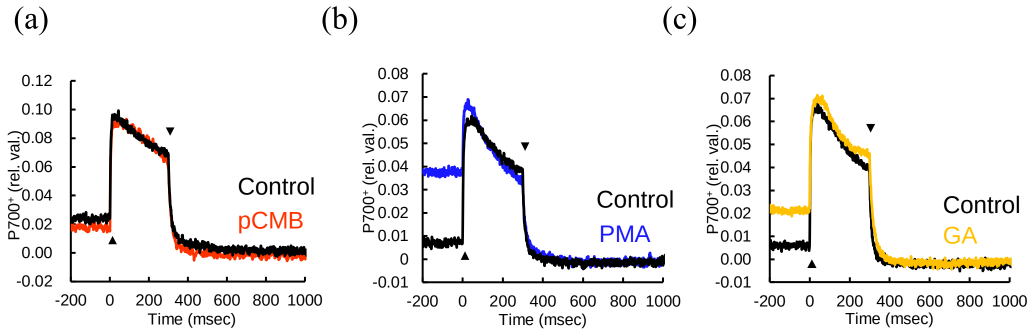

Supplementary Figure 12. Effects of photosynthesis inhibitors on the kinetics of P700 oxidation. Slr0688i cells (24 µg chl/ml) were treated with (a) pCMB (red) for 1.5 h, (b) 50 µM PMA (blue) for 30 min, and (c) 10 mM GA (yellow) for 30 min in BG11, followed by P700 measurements. Black lines are the control traces obtained with cell suspensions incubated with respective volumes of solvents without inhibitors. Far-red (FR) light was illuminated for 10 sec in advance, followed by the irradiation of multiple-turnover (MT) flash of which the beginning is indicated by upward black triangles; FR light and MT flash were then simultaneously turned off at the time indicated by downward black triangles. Source data are provided as a Source Data file.

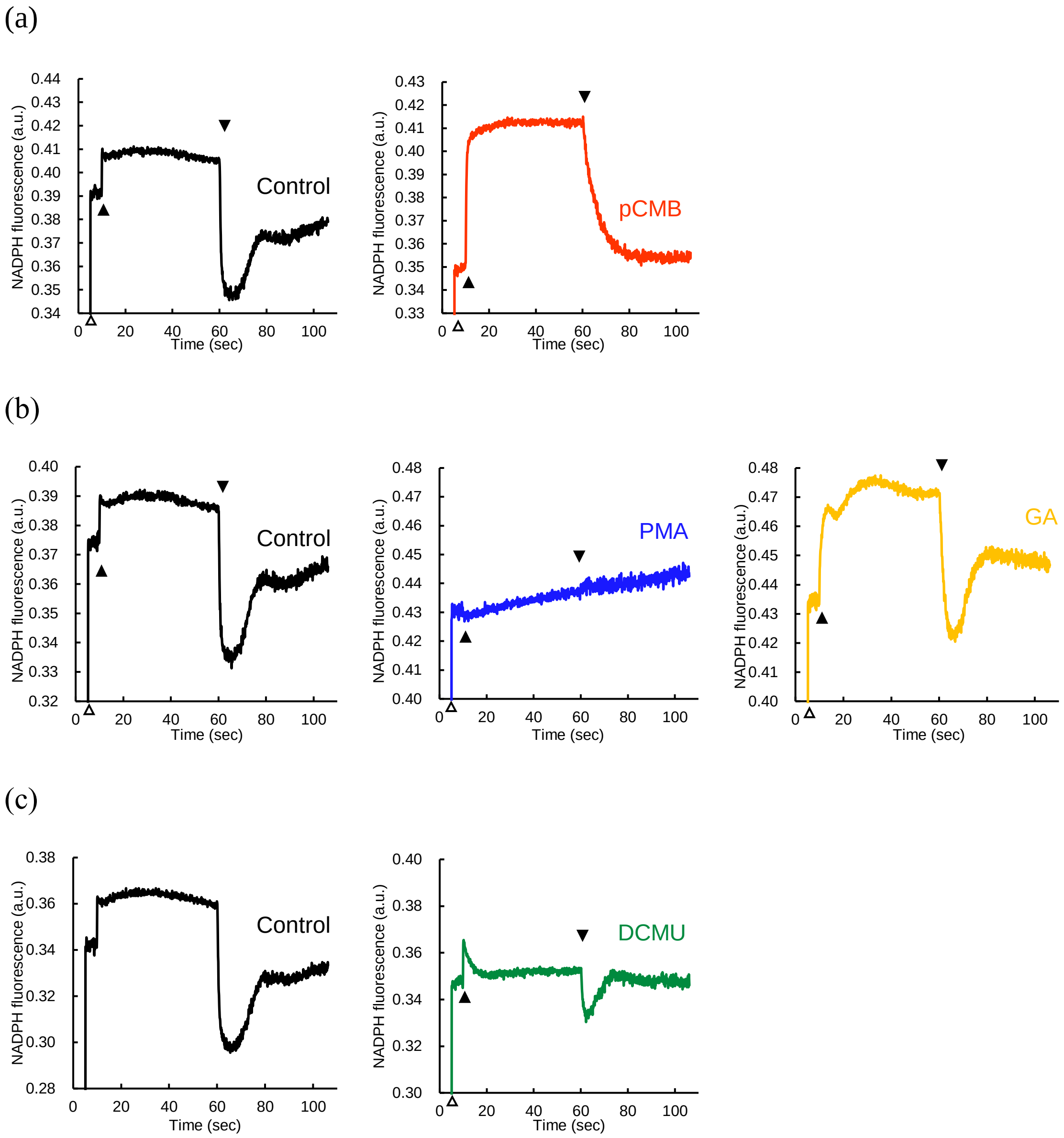

Supplementary Figure 13. Effects of photosynthesis inhibitors on changes in NADPH fluorescence. NADPH generation upon illumination was monitored with slr0688i cells treated with the photosynthesis inhibitors. Slr0688i cells (24 µg chl/ml) in BG11 were treated with (a) pCMB (red) for 1.5 h, (b) 50 µM PMA (blue), 10 mM GA (yellow) for 30 min in the dark, followed by dilution to 2.4 µg chl/ml and measurements. (c) 10 µM DCMU (green) was added to slr0688i cells (2.4 µg chl/ml) and incubated for 5 min, followed by measurements. The control datasets obtained with cell suspensions incubated with respective volumes of solvents without inhibitors are shown in black. The onset of the measuring light illumination is indicated by white upward triangles; the beginning and the end of the actinic light illumination are indicated by upward and downward black arrows, respectively. Source data are provided as a Source Data file.

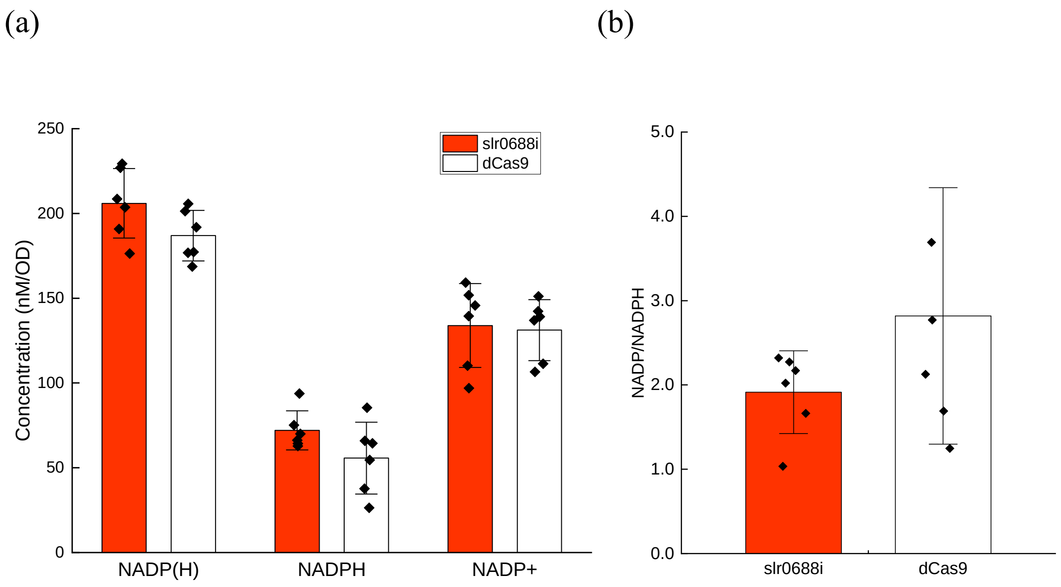

Supplementary Figure 14. Intracellular amounts of NADP(H) in slr0688i and dCas9. (a) NADP(H) contents in slr0688i (red bars) and dCas9 (white bars). (b) $NADP^+$/NADPH ratios calculated from NADP(H) contents. Presented are all the data points obtained (black diamonds) as well as average values of six biological replicates (bars) ± SD. Source data are provided as a Source Data file.

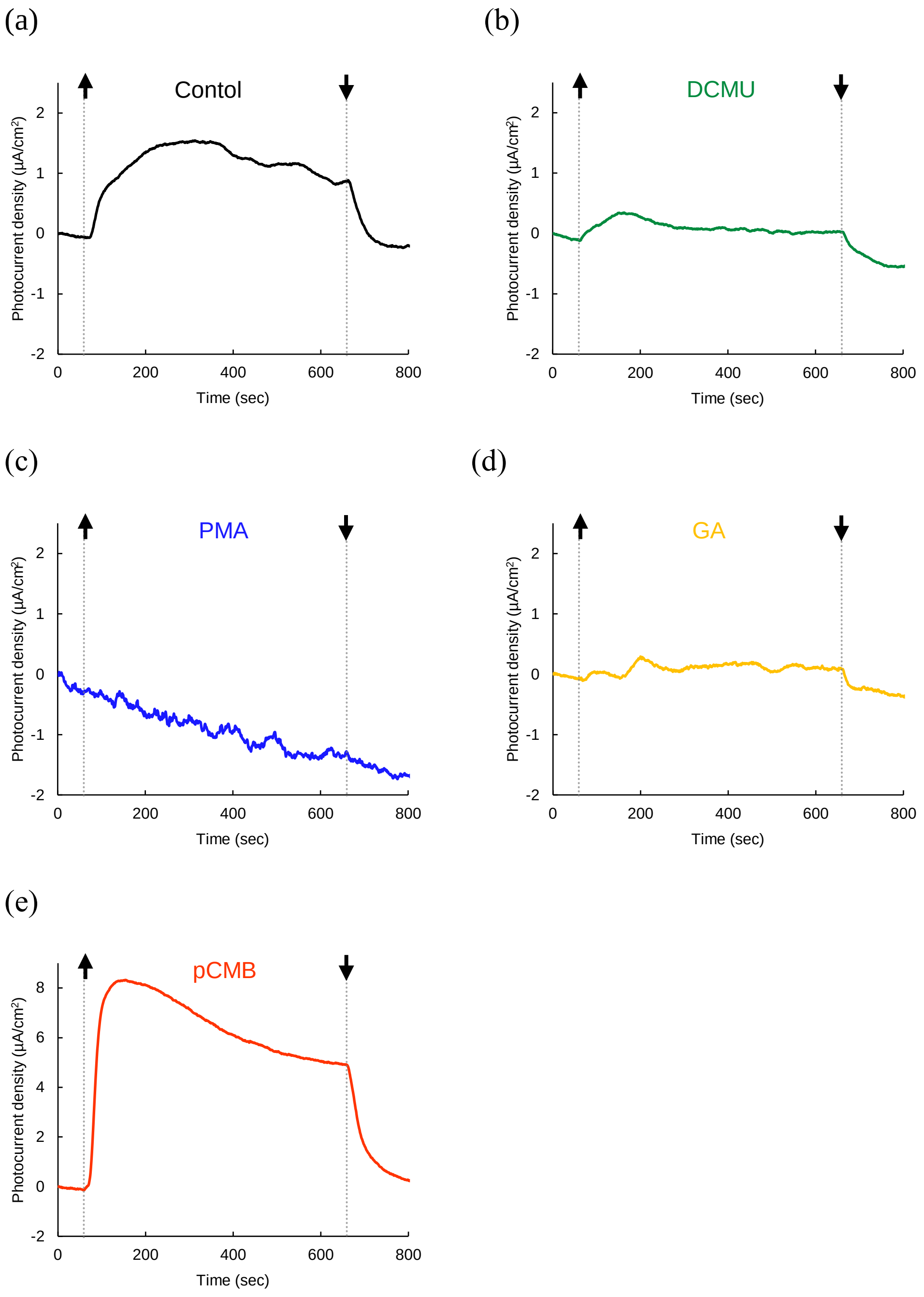

Supplementary Figure 15. Effects of photosynthesis inhibitors on photocurrent generation from slr0688i with a CP anode. Slr0688i cells (OD_{730} = 1.5, 4 mL) after 3 days of culture in supernatant were treated with (a) only solvent (black), (b) 10 µM DCMU (green), (c) 50 µM PMA (blue), (d) 10 mM GA (yellow) and (e) 100 µM pCMB (red), followed by injection by gravity onto a piece of carbon paper placed upon plane

ITO. Current before light illumination was set to zero, and photocurrent generated at +0.25 V vs. Ag/AgCl are shown. The upward and downward arrowheads indicate the beginning and end of illumination (420 μmol photons m^{-2} s^{-1}), respectively. Source data are provided as a Source Data file.

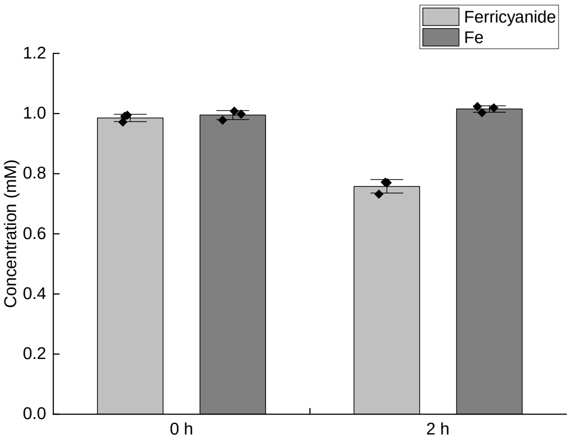

Supplementary Figure 16. Changes in concentration of ferricyanide and Fe element during ferricyanide assay. Changes in the Fe concentration during ferricyanide reduction assay were investigated. Slr0688i cells (OD_{730} = 1.0) resuspended with Fe-free BG11 were mixed with 1 mM potassium ferricyanide and incubated under illumination (50 µmol photons m^{-2} s^{-1}). The concentration of ferricyanide (light gray bars) and Fe (dark gray bars) in filtrated supernatants were measured with a spectrophotometer and ICP-MS, respectively, before (t = 0) and after (t = 2 h) the incubation. All the data points obtained (black diamonds) as well as average values of three biological replicates (bars) ± SD are shown. Source data are provided as a Source Data file.

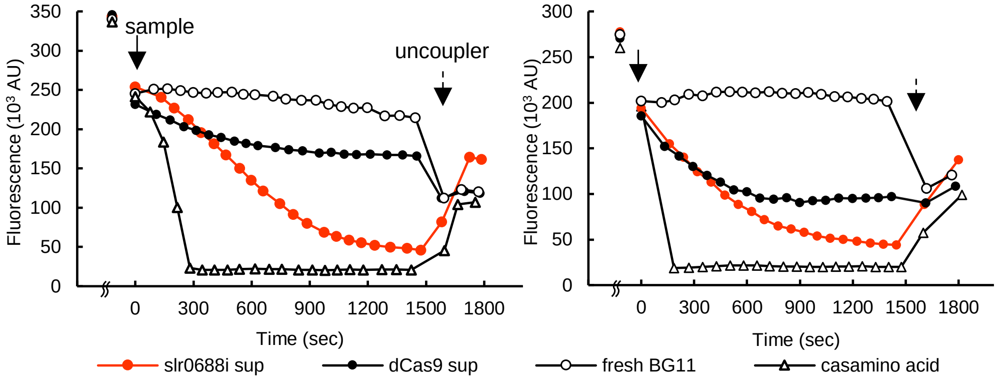

Supplementary Figure 17. Generation of membrane potential of *Bacillus* cells by supernatant of slr0688i. Shown are biological replicates of Fig. 4a. Changes in membrane potential of *Bacillus* cells were monitored following addition of each sample at the time indicated by bold arrows (t = 0): 5× concentrated supernatants with MW > 3,000 of slr0688i (red circles) and dCas9 (black circles), fresh BG11 (white circles), and 0.01% (w/v) casamino acid in BG11 (white triangles). At the time indicated by dotted arrows, 5 µM gramicidin was added to dissipate the membrane potential. Source data are provided as a Source Data file.

(a)

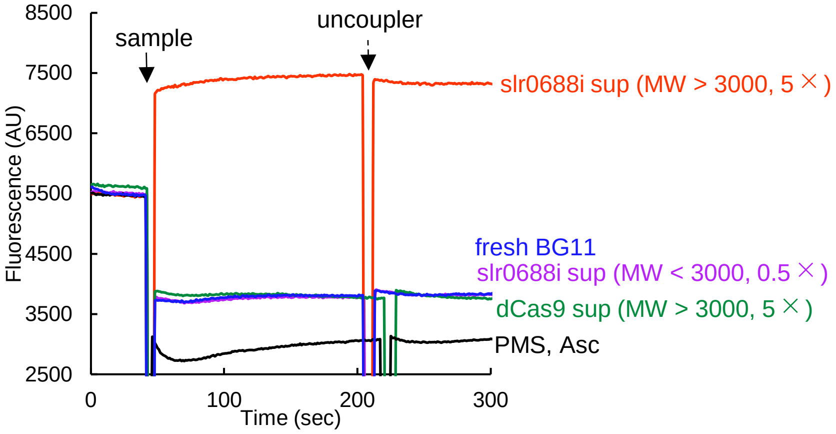

(b)

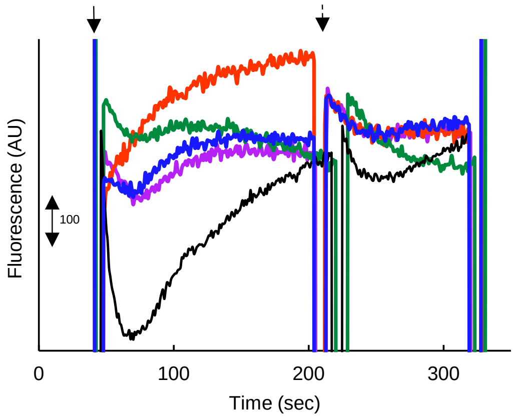

Supplementary Figure 18. The effect of slr0688i supernatant on changes in membrane potential of *Bacillus* membrane vesicles. Changes in membrane potential of membrane vesicles isolated from *Bacillus subtilis* (73 µg protein / mL, 0.9 mL) were monitored following addition of each sample (0.9 mL) at the time indicated by bold arrows: 5× concentrated supernatants with MW > 3,000 of slr0688i (red) and dCas9 (green), a 0.5× concentration of supernatants with MW < 3,000 of slr0688i (violet), fresh BG11 (blue), and 2.5 µM PMS / 10 mM ascorbate in BG11 (black). For better comparison, traces shown in (a) are enlarged and normalized in (b). The measurements were performed following the procedures of the experiments with living *Bacillus* cells. Source data are provided as a Source Data file.

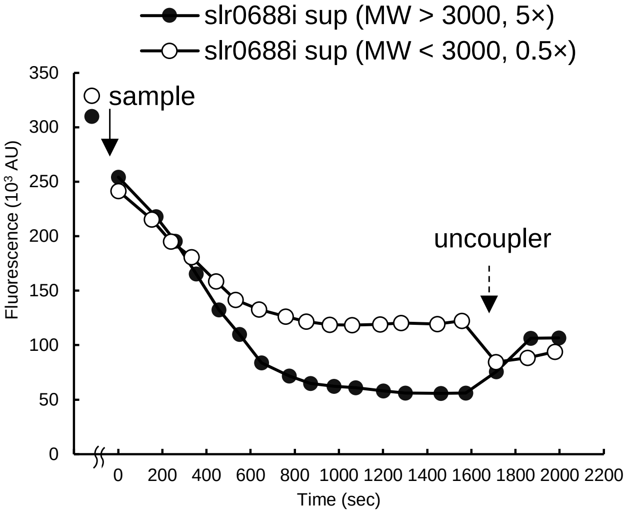

Supplementary Figure 19. Generation of membrane potential of *Bacillus* cells by size-fractionated supernatant of slr0688i. Changes in membrane potential of *Bacillus* cells were monitored following addition of a 5× concentrated supernatant with MW > 3,000 of slr0688i (black circles) and a 0.5× concentration of supernatant with MW < 3,000 of slr0688i (white circles). Each sample was added at the time indicated by a bold arrow (t = 0). At the time indicated by dotted arrows, 4 μM valinomycin was added to dissipate the membrane potential. Source data are provided as a Source Data file.

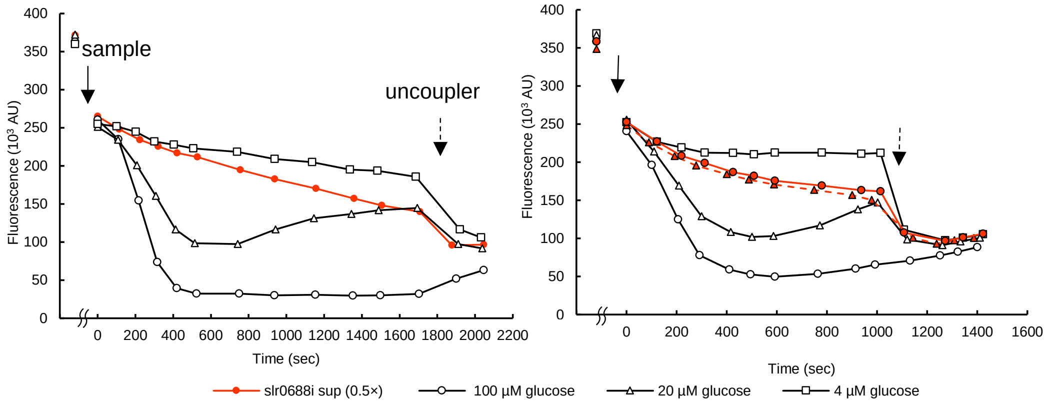

Supplementary Figure 20. Generation of membrane potential of *Bacillus* cells by supernatant of slr0688i. Shown are biological replicates of Fig. 4b. Changes in membrane potential of *Bacillus* cells were monitored following addition of each sample at the time indicated by bold arrows ($t = 0$): a 0.5× concentration of slr0688i supernatant (red circles and triangles), 100 µM (white circles), 20 µM (white triangles), 4 µM (white squares) glucose in BG11. At the time indicated by dotted arrows, 4 µM valinomycin was added to dissipate the membrane potential. Source data are provided as a Source Data file.

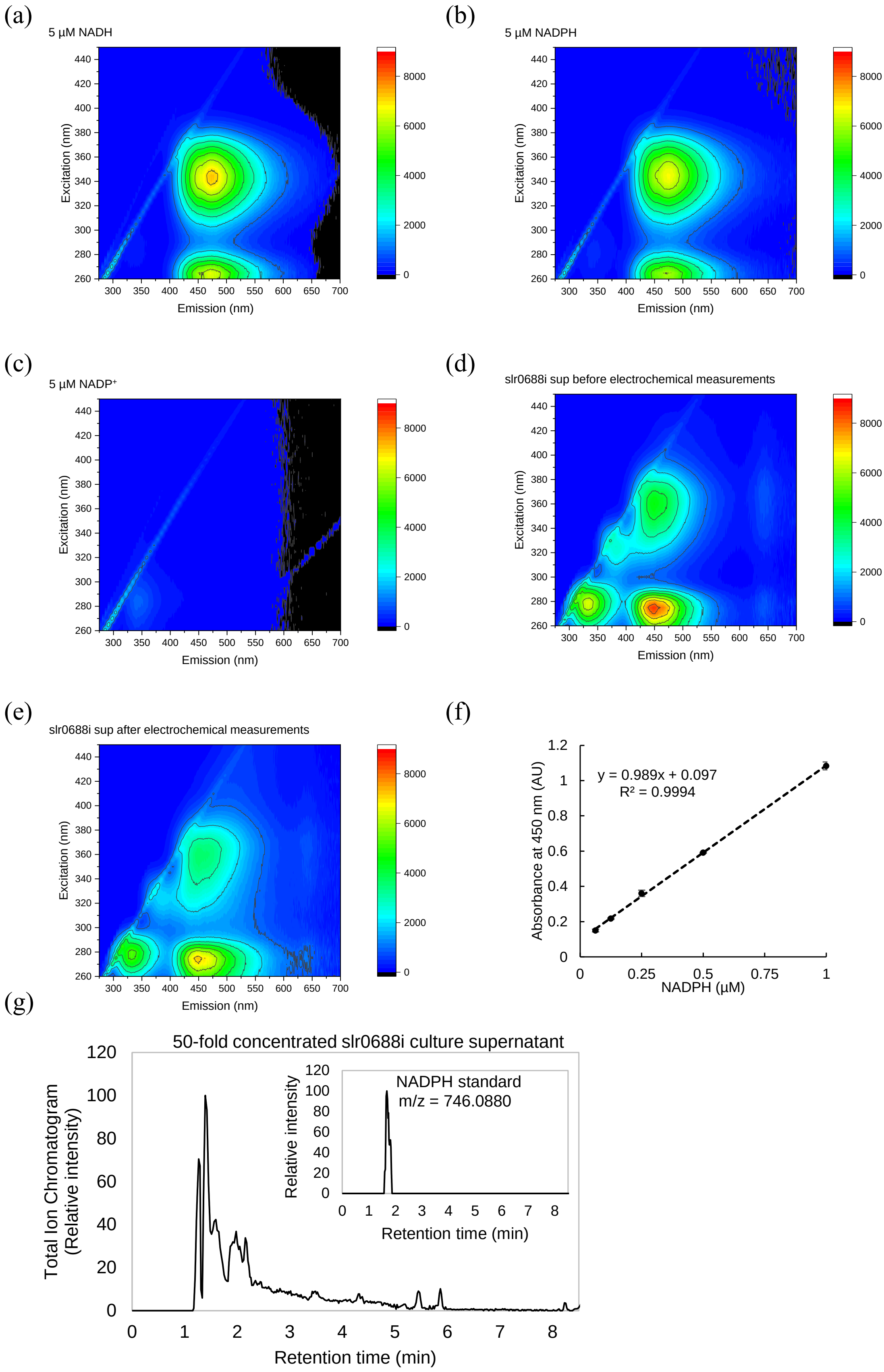
(a)
5 µM NADH
Excitation (nm)
Emission (nm)
(b)
5 µM NADPH
Excitation (nm)
Emission (nm)
(c)
5 µM NADP⁺
Excitation (nm)
Emission (nm)
(d)
slr0688i sup before electrochemical measurements
Excitation (nm)
Emission (nm)
(e)
slr0688i sup after electrochemical measurements
Excitation (nm)
Emission (nm)
(f)
y = 0.989x + 0.097
R² = 0.9994
Absorbance at 450 nm (AU)
NADPH (µM)
(g)
50-fold concentrated slr0688i culture supernatant
Total Ion Chromatogram (Relative intensity)
Retention time (min)
NADPH standard
m/z = 746.0880
Relative intensity
Retention time (min)

Supplementary Fig 21. Confirmation of the absence of NADPH in supernatants of slr0688i. (a-e) Two-dimensional fluorescence maps (2D-FMs) of (a) 5 μM NADH, (b) 5 μM NADPH, (c) 5 μM $NADP^+$ dissolved in BG11 and supernatants of slr0688i (d) before and (e) after electrochemical measurements with CP anodes. The supernatants were diluted to 0.1× concentration with BG11 before 2D-FM measurements. The color bars indicate the absolute values of fluorescence intensity. (f) An example of the calibration curve of NADP(H) enzymatic quantification assay. The absorbance at 450 nm (A_{450}), originating from WST formazan dye, was measured to quantify NADPH. A_{450} values of the supernatants, obtained either before or after electrochemical measurements with CP anodes, were lower than the detection limit of this assay, i.e., 0.0625 μM NADPH. Shown are average values of three technical replicates ± SD; note that the standard curve is generated every time the assay is performed and is reproducible. (g) Liquid chromatograph-mass spectrometry total ion chromatogram (TIC) of 50-fold concentrated slr0688i culture supernatant. The inset shows extracted ion chromatogram of m/z = 746.0880 of 25 μM NADPH standard solution. Whereas NADPH (m/z 746.0880) was detected as a peak at retention time 1.8 min in the standard solution, no peak at m/z ranging from 700 to 900 was found in the concentrated slr0688i supernatant. Source data are provided as a Source Data file.

(a)

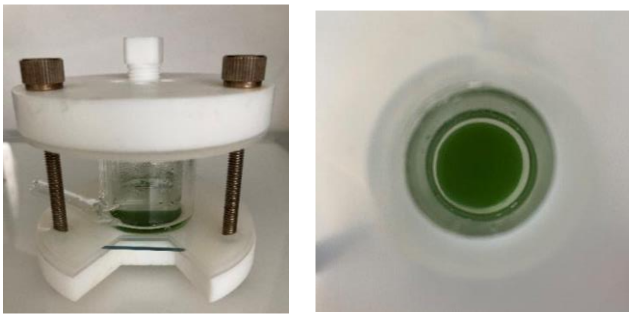

(b)

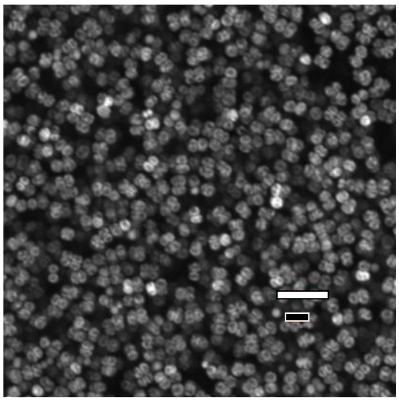

(c)

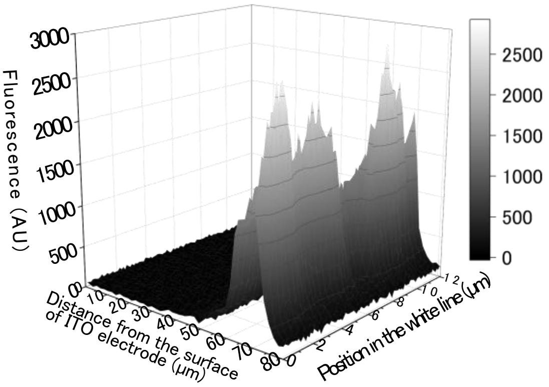
Fluorescence (AU)
3000
2500
2000
1500
1000
500
0
Distance from the surface of ITO electrode (μm)
0 10 20 30 40 50 60 70 80
Position in the white line (μm)
0 2 4 6 8 10 12
2500
2000
1500
1000
500
0

(d)

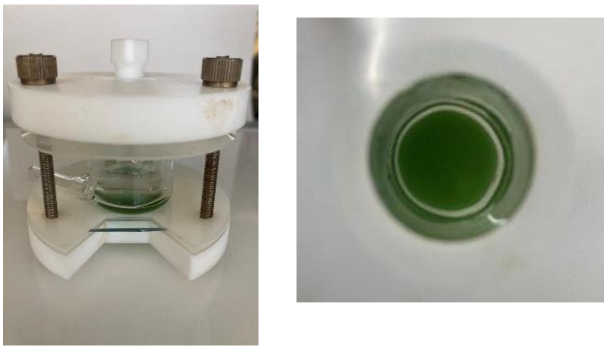

(e)

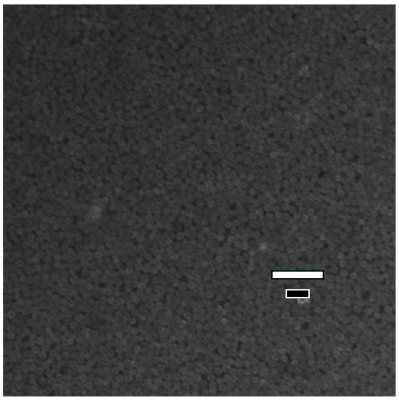

(f)

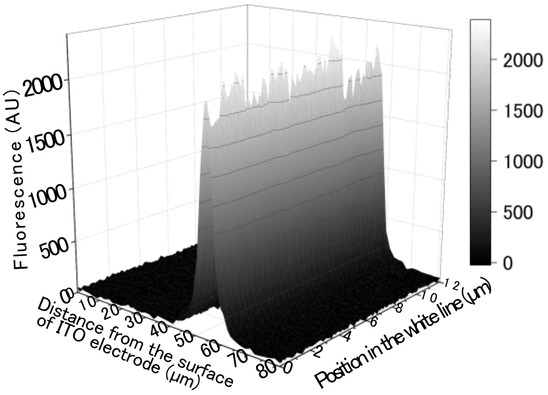
Fluorescence (AU)
2000
1500
1000
500
0
Distance from the surface of ITO electrode (μm)
0 10 20 30 40 50 60 70 80
Position in the white line (μm)
0 2 4 6 8 10 12
2000
1500
1000
500
0

Supplementary Figure 22. Photographs and confocal laser scanning microscopy (CLSM) images of *Synechocystis* cells. (a, d) Shown are photographs taken from the side (left) and the top (right) of slr0688i (a) and dCas9 (d) cell cultures (OD_{730} = 1.5, 4 mL) injected by gravity on flat ITO electrodes in electrochemical chambers. Each experiment was repeated 2 times independently, yielding similar results.

(b, e) Shown are CLSM images of slr0688i (b) and dCas9 (e) cells on flat ITO electrodes. Autofluorescence emission from the cells were recorded using CLSM. Black bars = 5 µm. These are the representative micrographs obtained by the observation of two biologically independent sample preparations.

(c, f) The intensity of autofluorescence was measured along the white lines shown in (b) and (e). Source data are provided as a Source Data file.

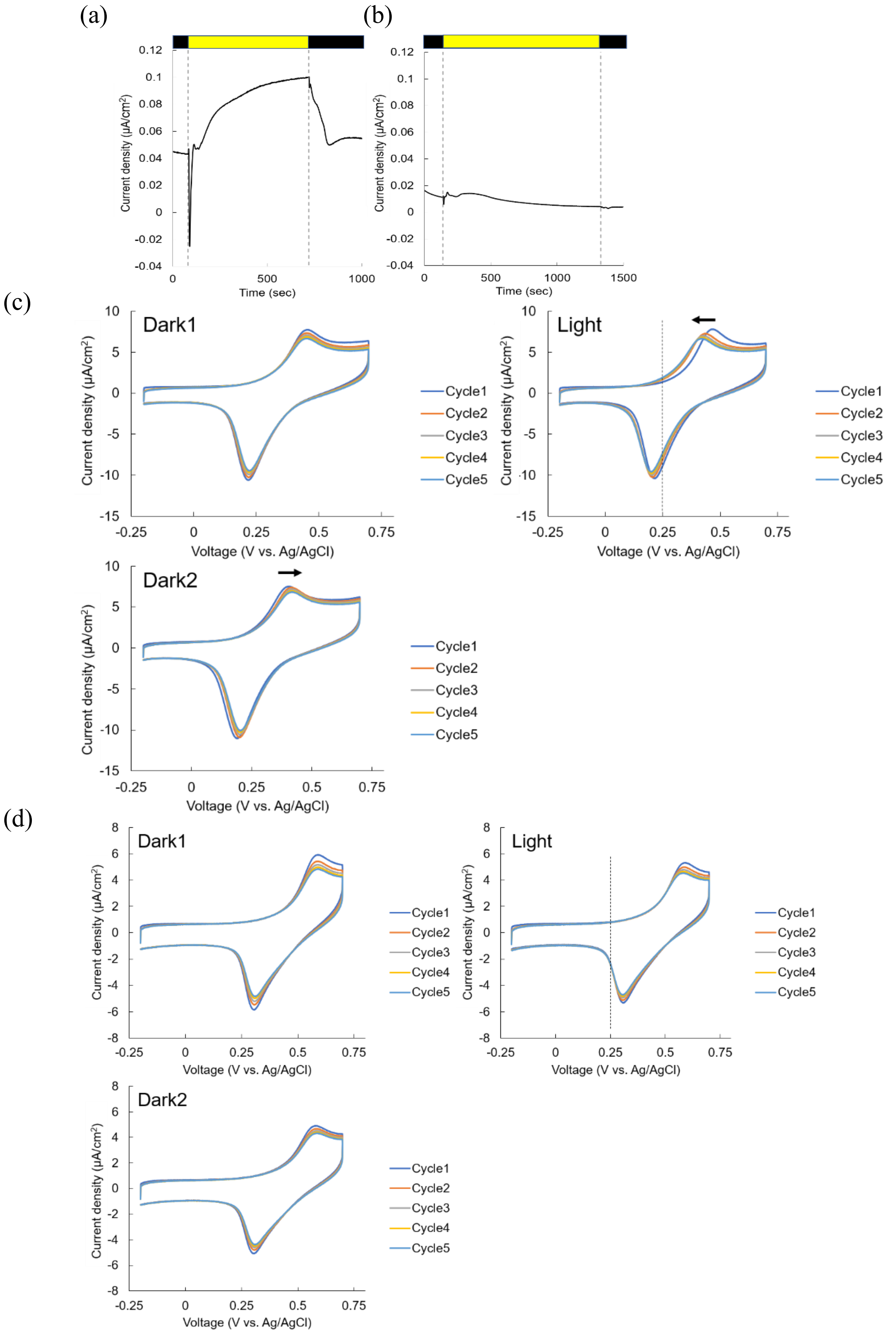
(a)
(b)
Current density (μA/cm²)
Time (sec)
(c)
Dark1
Light
Dark2
Current density (μA/cm²)
Voltage (V vs. Ag/AgCl)
Cycle1
Cycle2
Cycle3
Cycle4
Cycle5
(d)
Dark1
Light
Dark2
Current density (μA/cm²)
Voltage (V vs. Ag/AgCl)
Cycle1
Cycle2
Cycle3
Cycle4
Cycle5

Supplementary Figure 23. The effect of pH of cell suspensions on pseudo-photocurrent generation. Current generation from slr0688i was examined either (a, c) without adjusting pH (pH 8.6) or (b, d) after adjusting pH to 7.86 by addition of HCl. (a,c) The cell suspension with high pH (8.6) generated pseudo-photocurrent in the chronoamperogram at +0.25V vs Ag/AgCl due to an increase in pH under illumination, which is visualized in the cyclic voltammogram by shifts in the anodic peak potentials (indicated by black arrows in 'Light' and 'Dark2' conditions) attributed to manganese. (b,d) When pH of the cell suspension was adjusted to below 8, which is within buffering capacity of TES (pH 6.8~8.2), neither pseudo-photocurrent nor shifts in the anodic peak potentials were observed. The black and yellow rectangles in chronoamperograms a-b indicate dark and light (100 μmol photons m^{-2} s^{-1}) conditions, respectively. The dotted black lines in cyclic voltammograms c-d indicate +0.25V vs Ag/AgCl. Source data are provided as a Source Data file. Scan rate, 50 mV sec

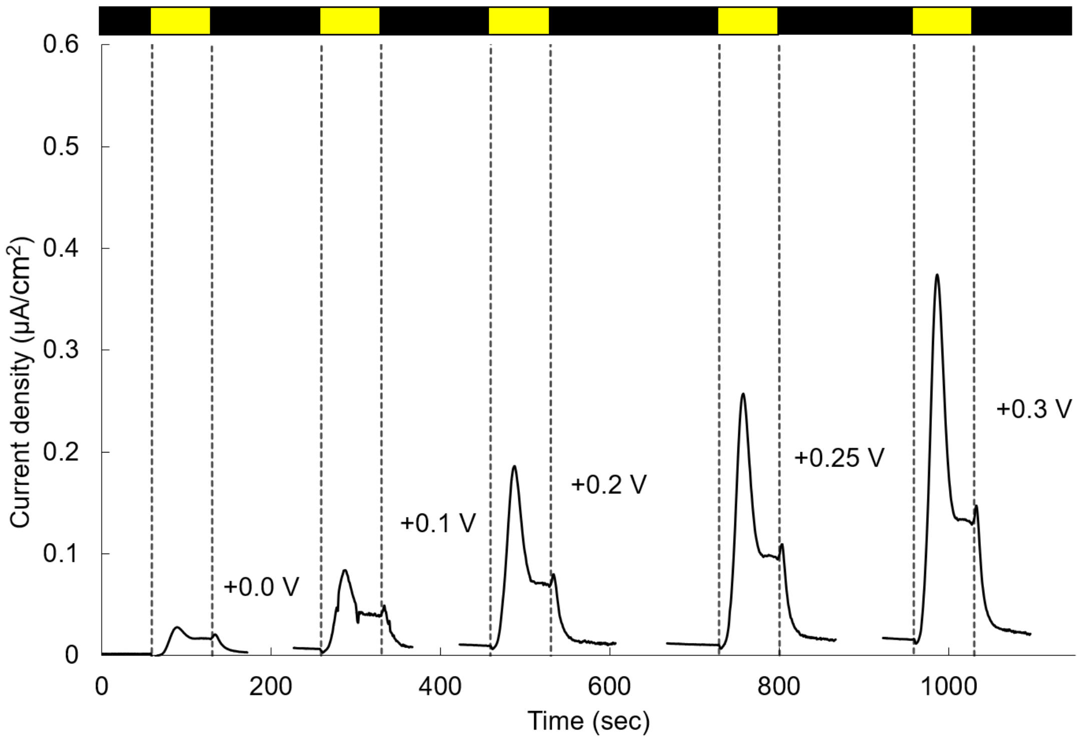

Supplementary Figure 24. Photocurrent generation from slr0688i at various voltages. Slr0688i (OD_{730}=2.7) was tested for its capacity to generate photocurrent at various voltages. The applied voltage was stepped from +0.0 V to +0.3 V vs Ag/AgCl. The black and yellow rectangles indicate dark and light (120 μmol photons m^{-2} s^{-1}) conditions, respectively. Source data are provided as a Source Data file.

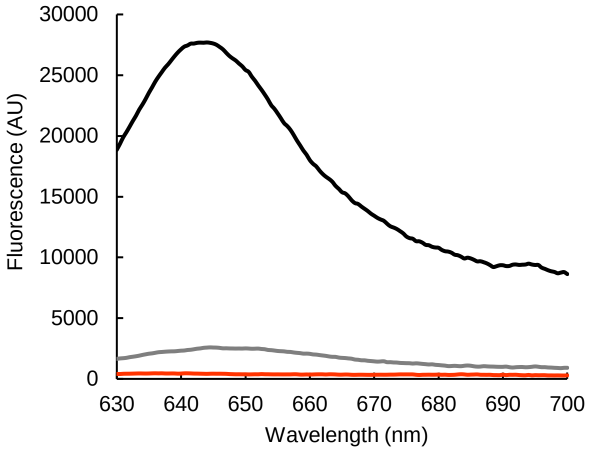

Supplementary Figure 25. Fluorescence spectra of 5× concentrated supernatants of slr0688i. Shown are fluorescence spectra of non-treated (black), O_2-purged (gray), and heat-treated (red) 5× concentrated slr0688i supernatants. Source data are provided as a Source Data file.

Supplementary Table 1. Current generated by slr0688i and dCas9 cells on ITO.

Time (sec)	Current density (μA/cm^2)	
	**Slr0688i**	**dCas9**
52	0.04 ± 0.03	0.003 ± 0.002
64	0.09 ± 0.06	0.002 ± 0.002
76	0.08 ± 0.04	0.003 ± 0.004
88	0.07 ± 0.04	0.003 ± 0.004
100	0.07 ± 0.04	0.003 ± 0.005

Averaged current density values ± 2SE at t = 52, 64, 76, 88 and 100 sec in Fig. 1b are presented.
