# Supplementary material for: Order-of-magnitude enhancement in photocurrent generation of Synechocystis sp. PCC 6803 by outer membrane deprivation
Source: Nat Commun. 2022 Jun 2;13:3067. doi: 10.1038/s41467-022-30764-z (PMC9163127; doi:10.1038/s41467-022-30764-z)
Supplement: Supplementary file 3 — Reporting Summary [file 41467_2022_30764_MOESM3_ESM.pdf]

nature portfolio

Corresponding author(s): Seiji Kojima and Shuji Nakanishi

Last updated by author(s): May 9, 2022

Reporting Summary

Nature Portfolio wishes to improve the reproducibility of the work that we publish. This form provides structure for consistency and transparency in reporting. For further information on Nature Portfolio policies, see our Editorial Policies and the Editorial Policy Checklist.

Statistics

For all statistical analyses, confirm that the following items are present in the figure legend, table legend, main text, or Methods section.

n/a	Confirmed	
☐	☒	The exact sample size (*n*) for each experimental group/condition, given as a discrete number and unit of measurement
☐	☒	A statement on whether measurements were taken from distinct samples or whether the same sample was measured repeatedly
☐	☒	The statistical test(s) used AND whether they are one- or two-sided<br>*Only common tests should be described solely by name; describe more complex techniques in the Methods section.*
☐	☒	A description of all covariates tested
☐	☒	A description of any assumptions or corrections, such as tests of normality and adjustment for multiple comparisons
☐	☒	A full description of the statistical parameters including central tendency (e.g. means) or other basic estimates (e.g. regression coefficient) AND variation (e.g. standard deviation) or associated estimates of uncertainty (e.g. confidence intervals)
☐	☒	For null hypothesis testing, the test statistic (e.g. *F*, *t*, *r*) with confidence intervals, effect sizes, degrees of freedom and *P* value noted<br>*Give P values as exact values whenever suitable.*
☒	☐	For Bayesian analysis, information on the choice of priors and Markov chain Monte Carlo settings
☒	☐	For hierarchical and complex designs, identification of the appropriate level for tests and full reporting of outcomes
☒	☐	Estimates of effect sizes (e.g. Cohen's *d*, Pearson's *r*), indicating how they were calculated

Our web collection on statistics for biologists contains articles on many of the points above.

Software and code

Policy information about availability of computer code

Data collection	ALS/CHI electrochemical analyzer Version 14.01J; GL220_820APS Version 1.12; OxyTrace+ Windows software Version 1.0.48; Spectra Manager Version 2.12.00; Dual PAM Version 3.12(Windows 10); PamWin Version 3.22d; FV10-ASW Version 04.02; LCMSsolution Version 3.80
Data analysis	Microsoft Excel for Microsoft 365 MSO Version 2202; FV10-ASW Version 04.02; LCMSsolution Version 3.80; OriginPro 2021b (64-bit) SR2 Version 9.8.5.212

For manuscripts utilizing custom algorithms or software that are central to the research but not yet described in published literature, software must be made available to editors and reviewers. We strongly encourage code deposition in a community repository (e.g. GitHub). See the Nature Portfolio guidelines for submitting code & software for further information.

Data

Policy information about availability of data

All manuscripts must include a data availability statement. This statement should provide the following information, where applicable:

- Accession codes, unique identifiers, or web links for publicly available datasets
- A description of any restrictions on data availability
- For clinical datasets or third party data, please ensure that the statement adheres to our policy

Figures that have associated raw data are Figs 1b-e, 2a, 3 and 4, and all the Supplementary Figs. The data that support the findings of the study are all available in the Source Data file.

March 2021

Field-specific reporting

Please select the one below that is the best fit for your research. If you are not sure, read the appropriate sections before making your selection.

- [x] Life sciences
- [ ] Behavioural & social sciences
- [ ] Ecological, evolutionary & environmental sciences

For a reference copy of the document with all sections, see nature.com/documents/nr-reporting-summary-flat.pdf

Life sciences study design

All studies must disclose on these points even when the disclosure is negative.

Sample size	Sample size was determined so that experiments were performed with at least 3 biological replicates.
Data exclusions	No data were excluded from the analysis.
Replication	All the experiments were repeated with at least 3 biological replicates, and all attempts at replication were successful.
Randomization	Sufficient biological replicates were prepared in all the experiments. In each biological replicate, different flasks containing different batch of cell cultures (always containing more than 1,000,000,000 cells/ml) were used for experiments; therefore, the collected data in this study are, essentially, already averaged and randomized, and randomization performed in clinical trials is not relevant to our study.
Blinding	Blinding was not performed in our study because 1) it was not practical to allocate two experimenters to each experiment and blind one of them handling many batches of microorganisms at one time, and 2) we recorded all the data obtained and analyzed all of them without any data exclusions or manipulations.

Reporting for specific materials, systems and methods

We require information from authors about some types of materials, experimental systems and methods used in many studies. Here, indicate whether each material, system or method listed is relevant to your study. If you are not sure if a list item applies to your research, read the appropriate section before selecting a response.

Materials & experimental systems

n/a	Involved in the study	
x		Antibodies
x		Eukaryotic cell lines
x		Palaeontology and archaeology
x		Animals and other organisms
x		Human research participants
x		Clinical data
x		Dual use research of concern

Methods

n/a	Involved in the study	
x		ChIP-seq
x		Flow cytometry
x		MRI-based neuroimaging
